# Exosomes secreted from bone marrow mesenchymal stem cells suppress cardiomyocyte hypertrophy through Hippo-YAP pathway in heart failure

**DOI:** 10.1590/1678-4685-GMB-2022-0221

**Published:** 2023-03-13

**Authors:** Yu Ren, Yun Wu, Wenshuai He, Yingjie Tian, Xingsheng Zhao

**Affiliations:** 1 Inner Mongolia People’s Hospital, Department of Scientific Research, Hohhot, China. Inner Mongolia People’s Hospital Department of Scientific Research Hohhot China; 2 Inner Mongolia People’s Hospital, Department of Cardiology, Hohhot, China. Inner Mongolia People’s Hospital Department of Cardiology, Hohhot China; 3 Inner Mongolia People’s Hospital, Clinical Medical Research Center in Cardiovascular Diseases, Hohhot, China. Inner Mongolia People’s Hospital Clinical Medical Research Center in Cardiovascular Diseases Hohhot China

**Keywords:** Mesenchymal stem cells, exosomes, cardiomyocyte hypertrophy, heart failure, Hippo-YAP pathway

## Abstract

Mesenchymal stem cells-derived exosomes (MSCs-exosomes) reportedly possess cardioprotective effects. This study investigated the therapeutic potential and mechanisms of MSCs-exosomes on heart failure (HF). H9c2 cells were used to establish a cardiomyocyte hypertrophy model by angiotensin II (Ang II) treatment. Isolated MSCs-exosomes were identified by transmission electron microscope and CD63 detection. Apoptosis rate was measured by terminal deoxynucleotidyl transferase (TdT) dUTP Nick-End Labeling (TUNEL) assay. Levels of inflammatory factors [interleukin (IL)-1β, IL-4, IL-6, and tumor necrosis factor (TNF)-α] and brain natriuretic peptide (BNP) were determined by ELISA. Expression of apoptosis-related proteins [Bax, B-cell lymphoma-2 (Bcl-2), and caspase 3] and Hippo-Yes-associated protein (YAP) pathway-related proteins [YAP, phosphor (p)-YAP, and tafazzin (TAZ)] was detected by western blotting. Cardiomyocyte hypertrophy of H9c2 cells induced by Ang II was ameliorated by MSCs-exosomes treatment. MSCs-exosomes downregulated Bax and caspase 3 levels and upregulated Bcl-2 level in Ang II-induced H9c2 cells. MSCs-exosomes also reduced the levels of BNP, IL-1β, IL-4, IL-6, and TNF-α in Ang II-induced H9c2 cells. Meanwhile, p-YAP was downregulated and TAZ was upregulated after MSCs-exosomes administration. In conclusion, MSCs-exosomes alleviate the apoptosis and inflammatory response of cardiomyocyte via deactivating Hippo-YAP pathway in HF.

## Introduction

Heart failure (HF) is a common clinical syndrome characterized by dyspnea, fatigue, and volume overload finally leading to death, which can be caused by various cardiovascular diseases (Tuttolomondo et al., 2016). Even though there has been a breakthrough in therapy during the past three decades, the survival rate of patients with HF is still at a dismal 50%, and 300,000 people suffer from HF per year (Epstein et al., 2011). Currently, heart transplantation is the main therapeutic approach for HF; however, heart transplantation recipient will take risks of transplant organ rejection and complications (Martinez et al., 2019; Wang et al., 2020). Therefore, finding an effective therapeutic approach for HF is urgently needed. 

Cardiomyocyte hypertrophy is the basic pathological characteristic of HF, which is accompanied with inflammation and apoptosis (Hashimoto et al., 2018). Therefore, it is critical to prevent hypertrophic progress of cardiomyocytes in HF. Numerous researchers found that mesenchymal stem cells (MSCs) as multipotent and undifferentiated cells can limit cardiac hypertrophy, apoptosis, and inflammation (Li et al., 2008; Wen et al., 2011; Mokhtari et al., 2020; Philipp et al., 2021). MSCs exert beneficial effects on the heart by paracrine secretion, and exosome is one of the most important secretory products of MSCs (Tsao et al., 2014). Exosomes, a kind of bioactive vesicles, play an essential role in regulating cellular progresses, including proliferation, migration, and apoptosis (Wu et al., 2018). Reports have shown that MSCs-derived exosomes (MSCs-exosomes) can transfer lipids, proteins, and RNAs to damaged cardiomyocytes, thereby reducing cell apoptosis and inflammation in HF (Wang et al., 2021). Wen et al. (2020) indicated that bone marrow MSC-derived exosomes could reduce the hypoxia-induced apoptosis of cardiomyocytes. Liu et al. (2019) found that MSCs-exosomes could inhibit hypoxia and serum deprivation-induced cardiomyocytes apoptosis. Also, exosomes derived from bone marrow MSCs can repress cardiac fibrosis in rats with atrial fibrillation (Xu et al., 2022). However, the effects of MSCs-exosomes on pathological hypertrophic cardiomyocytes in HF remain unclear.

The Hippo-Yes-associated protein (YAP) pathway plays an important role in cardiac cell proliferation and apoptosis (Plouffe *et al.*, 2015; Zhang *et al.*, 2019). Chen *et al.* (2021) found that the enhancement of nuclear YAP abundance inhibits cardiomyocyte apoptosis, thereby reducing myocardial infarction. von Gise *et al.* (2012) indicated that the YAP deletion can cause lethal cardiac hypoplasia. However, it is still unclear whether the Hippo-YAP pathway is involved in the regulation of MSCs-exosomes in HF. 

In the present study, we explored the anti-inflammation and anti-apoptosis effects of MSCs-exosomes on hypertrophic cardiomyocytes induced by angiotensin II (Ang II). The possible mechanism of MSCs-exosomes against HF involving Hippo-YAP pathway was also unveiled. This research may provide the clinical basis for HF treatment using MSCs-exosomes.

## Material and Methods

### Animals

All experimental operation was performed according to the standard of the Institutional Animal Care and Research Advisory Committee of Inner Mongolia People’s Hospital (approval No. 2022LL003). Adult male Sprague-Dawley (SD) rats (180-220 g, n = 5) were purchased from the Animal Center of Wuhan University. All rats were maintained in a pathogen-free animal facility and provided water and food at 23 ± 1 ℃ with humidity of 55 ± 5%.

### Isolation and identification of MSCs

SD rats were anesthetized with 200 mg/kg of sodium pentobarbital and sacrificed by cervical dislocation. The femur and tibia without muscles were removed and washed with high glucose Dulbecco's modified Eagle medium (DMEM) (Gibco, MD, USA) for MSCs isolation. MSCs were incubated with DMEM containing 100 U/mL penicillin, 100 μg/mL streptomycin, and 10% fetal bovine serum (FBS) under a humidified condition at 37 ℃ with 5% CO_2_. Then, cell morphological observation was performed using an inverted microscope (Leica, Germany). In addition, the specific expression of MSCs markers (CD90, CD105, and CD34) was identified by flow cytometry (Beckman Coulter, CA, USA). The osteogenic and adipogenic differentiation abilities of MSCs were analyzed by alizarin red staining and oil red O staining (Solarbio, China), respectively.

### Isolation of exosomes from MSCs

The cultured MSCs were centrifuged twice at 500 ×*g* for 15 min, three times at 2,500 ×*g* for 15 min, and twice at 15, 000 ×*g* for 25 min. The supernatant fluid was transferred to Ultra-Clear tubes and then centrifuged at 80,000 ×*g* for 2 h at 4 °C. The exosomes-containing pellet was rinsed with phosphate buffered saline (PBS) and continued to centrifuge at 80,000 ×*g* for 1 h. The pellet was resuspended in 250 μL PBS and stored at −80 °C until use.

### Transmission Electron Microscopy (TEM)

MSCs-exosomes (10 μL) fixed with 2.0% glutaraldehyde (Merck, Germany) were dropped to the carbon coated copper grid for 90 s. After drying for 10 min, grids were incubated with 10 μL uranyl acetate (pH 7.0, SPI-CHEM, PA, USA) for 10 min. MSCs-exosomes were observed under a FEI Tecnai T20 transmission electron microscope (FEI, Netherlands) with 120 kV.

### Nanoparticle Tracking Analysis (NTA)

The Nanosight NS 300 system (NanoSight Technology, UK) was used to trace the size and quantity of MSCs-exosomes. Resuspensions of exosomes with PBS were further diluted 500-fold, and then artificially injected into the sample chamber at room temperature. MSCs-exosomes were detected under a complementary metal-oxide semiconductor camera with a setting of 488 nm and 30 s acquisition time. NTA analytical software (version 2.3) was used for data analysis.

### Cell culture and treatment

H9c2 cells (rat embryonic cardiomyocytes) were purchased from the American Type Culture Collection (ATCC, VA, USA). Cells were cultured in DMEM containing 10% FBS and 100 IU/mL penicillin-streptomycin. The incubation was maintained in a humid environment at 37 ℃ with 5% CO_2_. When cell conﬂuency reached 70%-80%, H9c2 cells were treated by graded concentrations of Ang II (0, 0.1, 0.2, 0.4, 0.8, 1, 2, 4, 8, and 10 μM) (Merck Millipore, Billerica, MA, USA) for 24 and 48 h to obtain the appropriate concentration to induce cell hypertrophy. H9c2 cells were divided into three groups: the negative control (NC) group (H9c2 cell without Ang II induction), the Ang II group, and the Ang II + exosomes (Exo) group. H9c2 cells in the Ang II group were treated with 0.4 μM Ang II for 24, 48, and 72 h. After Ang II induction, H9c2 cells in the Ang II + Exo group were co-cultured with MSCs-exosomes for 24, 48, and 72 h.

### Cell proliferation assay

The viability of H9c2 cells was detected by the Cell Counting Kit (CCK)-8 assay kit (TransGen Biotech, China). H9c2 cells at a density of 1 × 10^5^ cells/mL were seeded in a 96-well plate, and then the CCK-8 solution was added into each well and incubated for 24, 48, and 72 h. Absorbances at 450 nm were measured with a multifunctional microplate reader (Molecular Devices, CA, USA). The appropriate concentration of Ang II at 48 h was determined by the half-maximal inhibitory concentration (IC50) value calculated by GraphPad Prism 5.0.

### Enzyme-Linked Immunosorbent Assay (ELISA)

According to the manufacturer’s instructions, ELISA kits (Elabscience, China) were used for quantitative analysis of interleukin (IL)-6, IL-1β, tumor necrosis factor (TNF)-α, IL-4, and brain natriuretic peptides (BNP) in H9c2 cells after culturing 24 h. Levels of these parameters were determined using a DR-200Bs microplate reader at 450 nm (Diatek, China).

### Apoptosis test

The apoptosis of H9c2 cells was determined using terminal deoxynucleotidyl transferase (TdT) dUTP Nick-End Labeling (TUNEL; Roche, Mannheim, Germany) method under the instruction of the manufacturer. Briefly, 50 μL TUNEL reaction mixture was added to H9c2 cells (2 × 10^6^ cells/mL) and incubated for 1 h in a humidified and dark condition. Cell nuclei were stained by 4',6-diamidino-2-phenylindole (DAPI) showing blue fluorescence. The TUNEL (green fluorescence) positive cells were apoptotic H9c2 cells. The apoptosis was observed via a fluorescence microscope (NIKON TE200-U, Japan) and were counted from six randomly selected fields.

### Western blotting

Total protein was extracted from H9c2 cells and MSCs-exosomes by radio-immunoprecipitation assay (RIPA) lysis buffer (TaKaRa, Japan) with protease inhibitors (Roche, China). The concentrations of proteins were detected by a bicinchoninic acid (BCA) protein assay kit (Pierce, Netherlands). Sodium dodecyl sulphate-polyacrylamide gel electrophoresis (SDS-PAGE) was used to separate equal quantities of total proteins (20 μg per lane), and then separated proteins were transferred onto polyvinylidene difluoride membrane (Millipore, MA, USA). Membranes were blocked by PBS-5% fat-free dried milk at room temperature for 1 h and then incubated at 4 ℃ overnight with anti-CD63 (1:1,000), anti-YAP (1:2,000), anti-phosphor (p)-YAP (1:2,000), anti-tafazzin (TAZ) (1:2,000), anti-caspase 3 (1:1,000), anti-B-cell lymphoma-2 (Bcl-2) (1:2,000), anti-Bax (1:1,000), and anti- glyceraldehyde-3-phosphate dehydrogenase (GAPDH) (1:3,000) (Abcam, UK). Then, the goat anti-rabbit horseradish peroxidase-conjugated secondary antibody was incubated with membranes for 2 h. Protein bands were visualized by ChemiDoc XRS+ system (Bio-Rad, CA, USA).

### Statistical analysis

Data were analyzed via GraphPad Prism 5.0 and were presented as mean ± standard error of the mean (SEM). Comparisons between different groups were done by one-way analysis of variance (ANOVA) followed by unpaired 𝑡-test or a post hoc’ multiple comparison test. The family-wise significance and confidence level was set as 0.05 (95% confidence interval), that is P value < 0.05.

## Results

### Identification of MSCs and exosomes

**Figure 1 -:**
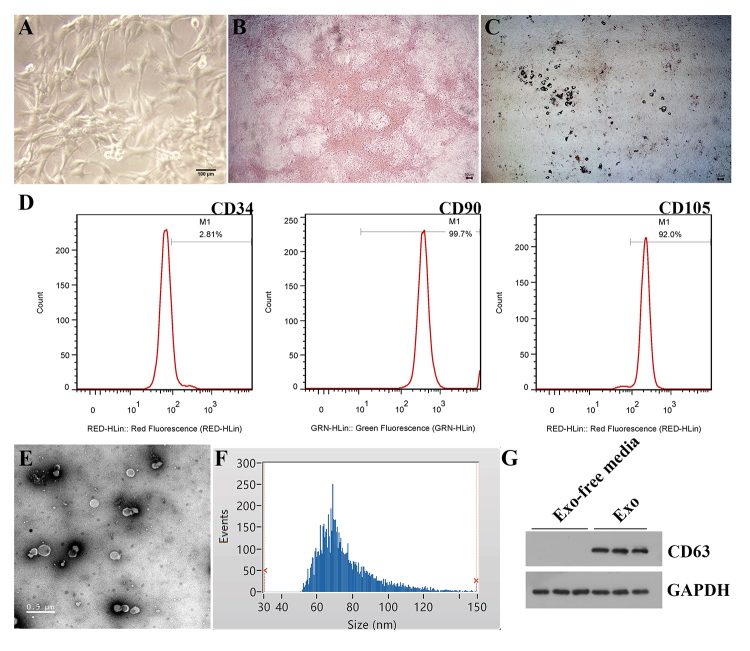
Characterization of mesenchymal stem cells (MSCs) and MSCs-exosomes. A. Morphological observation of MSCs. Scale bar = 100 μm. B. Alizarin red staining of MSCs. Scale bar = 50 μm. C. Oil red O staining of MSCs. Scale bar = 50 μm. D. The surface antibodies (CD34, CD90, and CD105) of MSCs were detected by flow cytometry. E. Morphology of MSCs-exosomes was observed by transmission electron microscopy. Scale bar = 0.5 μm. F. Particle size distribution of MSCs-exosomes was measured by nanoparticle tracking analysis. G. Western blot analysis of the exosomes surface marker (CD63).

MSCs were isolated from the femur and tibia of SD rats. As shown in Figure 1A, MSCs presented a fibroblast-like morphology that is the typical form of MSCs. Then, the osteogenic and adipogenic differentiation of MSCs was evaluated by alizarin red staining and oil red O staining, respectively. Alizarin red staining showed a large number of calcified nodules, and oil red O staining presented evident lipid vacuoles accumulation in MSCs (Figure 1B and 1C ). In addition, CD90, CD105, and CD34 are the typical surface biomarkers of MSCs. As expected, MSCs showed the positive expression of CD90 and CD105, while CD34 was negative expression (Figure 1D ).

Subsequently, exosomes secreted from MSCs were isolated. Results showed that MSCs-exosomes exhibited a round-shaped morphology with a size ranging from 50 to 150 nm (average size = 75.82 nm) (Figure 1E and 1F). In addition, we found that CD63 (an exosomes protein marker) was highly abundant in MSCs-exosomes (Figure 1G).

### MSCs-exosomes increase the viability of cardiomyocytes with hypertrophy

**Figure 2 -:**
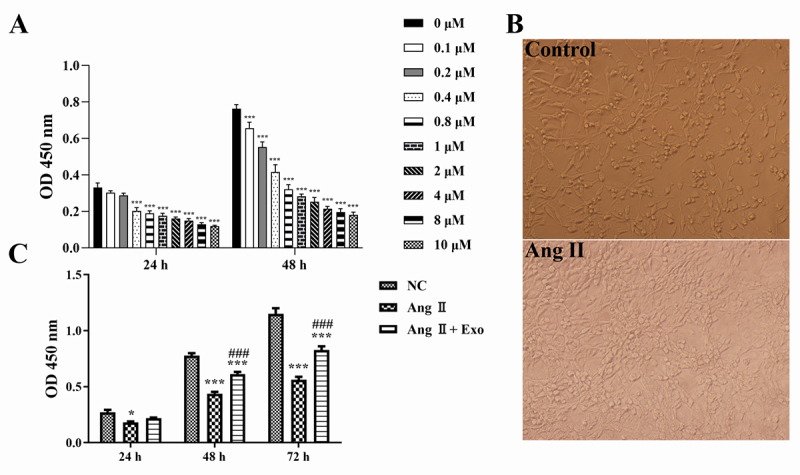
The effect of MSCs-exosomes on cardiomyocytes. A. The viability of H9c2 cells was examined by Cell Counting Kit (CCK)-8 assay. H9c2 cells were treated with different concentrations of angiotensin II (Ang II; 0-10 μM) for 24 and 48 h. B. Morphology of H9c2 cells treated with Ang II (0.4 μM). C. The proliferation of H9c2 cells was examined by CCK-8 assay. H9c2 cells were treated with Ang II (0.4 μM) or/and MSCs-exosomes. ^*^P < 0.05 and ^***^P < 0.001 vs. the NC (negative control) group; ^###^P < 0.001 vs. the Ang II group.

An in vitro model of cardiac hypertrophy was established in H9c2 cells by Ang II infusion (0-10 μM). CCK-8 assay showed that Ang II inhibited the viability of H9c2 cells in a dose-dependent manner (P < 0.001). After 48 h incubation, the IC50 value of Ang II was 0.4 μM (Figure 2A). Therefore, 0.4 μM Ang II was used to treat H9c2 cells for subsequent experiments. Compared with control cells, Ang II-treated cells were obviously hypertrophic (Figure 2B ).

Subsequently, we investigated the effect of MSCs-exosomes on hypertrophic cardiomyocytes. As shown in Figure 2C, H9c2 cell proliferation ability in the Ang II group was significantly lower than that in the NC group (P < 0.05). However, MSCs-exosomes addition dramatically enhanced the viability of H9c2 cells treated with Ang II (P < 0.001, Figure 2C).

### MSCs-exosomes inhibit inﬂammatory damage in hypertrophic cardiomyocytes

**Figure 3 -:**
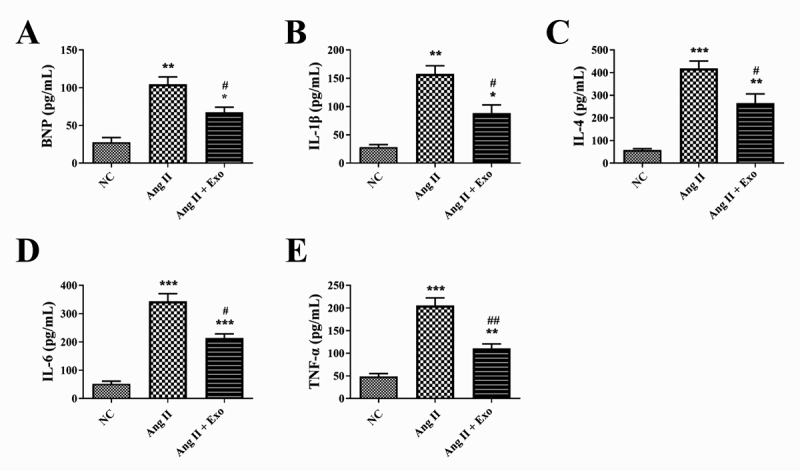
Effect of MSCs-exosomes on the inflammatory damage in hypertrophic cardiomyocytes. A-E. Levels of brain natriuretic peptide (BNP), interleukin (IL)-1β, IL-4, IL-6, and tumor necrosis factor (TNF)-α in H9c2 cells after treatment with Ang II or/and MSCs-exosomes. ^*^P < 0.05, ^**^P < 0.01, and ^***^P < 0.001 vs. the NC group; ^#^P < 0.05 and ^##^P < 0.01 vs. the Ang II group.

BNP is a cardiac hypertrophy marker protein (Ba et al., 2019). The concentration of BNP in H9c2 cells was increased by Ang II treatment, which was significantly reduced by MSCs-exosomes addition (P < 0.05, Figure 3A). In addition, the levels of inflammatory factors IL-1β, IL-4, IL-6, and TNF-α in H9c2 cells from the Ang II group were signiﬁcantly increased compared with that from the NC group (P < 0.01). MSCs-exosomes prominently inhibited the levels of IL-1β, IL-4, IL-6, and TNF-α in H9c2 cells with Ang II treatment (P < 0.05, Figure 3B-E ).

### Anti-apoptotic effect of MSCs-exosomes in hypertrophic cardiomyocytes

**Figure 4 -:**
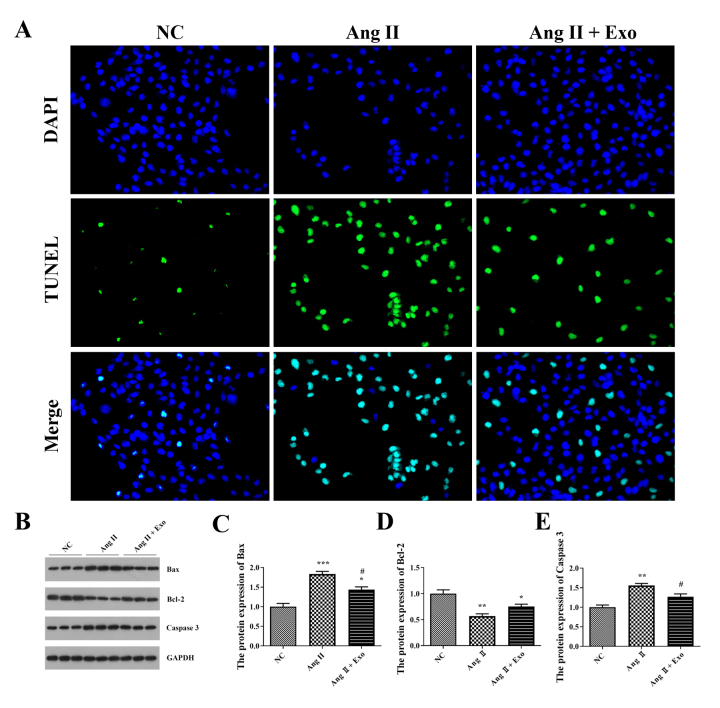
Anti-apoptosis effect of MSCs-exosomes on hypertrophic cardiomyocytes. A. The apoptosis test of H9c2 cells by terminal deoxynucleotidyl transferase (TdT) dUTP Nick-End Labeling (TUNEL) assay. Apoptotic cells were clearly identified with a strong nuclear green fluorescence (FITC). Cell nuclei were visualized as blue fluorescence (DAPI, 4’,6-diamidino-2-phenylindole). B-E. Expression levels of B-cell lymphoma-2 (Bcl-2), Bax, and caspase 3 in H9c2 cells were detected by western blotting. H9c2 cells were treated with Ang II or with Ang II + exosomes (Exo). ^*^P < 0.05, ^**^P < 0.01, and ^***^P < 0.001 vs. the NC group; ^#^P < 0.05 vs. the Ang II group.

To find out the biological functions of MSCs-exosomes on hypertrophic cardiomyocytes, the apoptosis of H9c2 cells was determined. TUNEL assay showed that Ang II treatment increased the apoptotic level of H9c2 cells compared with NC, while MSCs-exosomes addition decreased the apoptosis (Figure 4A). In addition, we examined the expression of pro-apoptotic proteins (Bax and caspase 3) and the anti-apoptotic protein Bcl-2 in H9c2 cells. As shown in Figure 4B-E, Ang II significantly promoted the expression of Bax and caspase 3, while inhibiting the expression of Bcl-2 in H9c2 cells (P < 0.01). The apoptosis level of Ang II-treated H9c2 cells was markedly alleviated after MSCs-exosomes addition, evidenced by the decreased Bax and caspase 3 levels, as well as the increased Bcl-2 level (P < 0.05, Figure 4B-E).

### MSCs-exosomes inhibits YAP signaling pathway in hypertrophic cardiomyocytes

**Figure 5 -:**
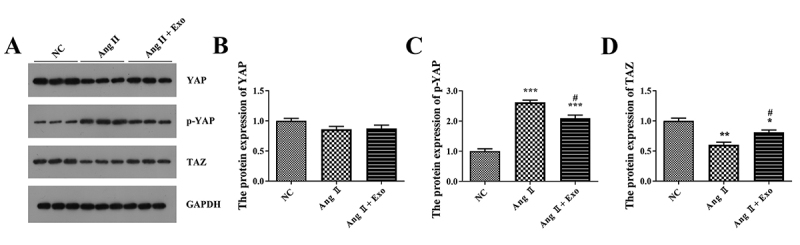
Hippo-Yes-associated protein (YAP) is involved in the effect of MSCs-exosomes on hypertrophic cardiomyocytes. A-D. Expression levels of YAP, phosphor (p)-YAP, and tafazzin (TAZ) in H9c2 cells were detected by western blotting. H9c2 cells were treated with Ang II or with Ang II + Exo. ^*^P < 0.05, ^**^P < 0.01, and ^**^P < 0.001 vs. the NC group; ^#^P < 0.05 vs. the Ang II group.

The Hippo-YAP signaling pathway is closely involved in the processes of heart development and regeneration (Zhao et al., 2011). YAP and TAZ are important regulating proteins in Hippo-YAP signaling. Here, the expression of p-YAP was increased in H9c2 cells from the Ang II group compared with that from the NC group (P < 0.001, Figure 5A-C ). MSCs-exosomes treatment decreased p-YAP expression in H9c2 cells treated with Ang II (P < 0.05). On the contrary, compared with the NC group, the expression of TAZ was reduced in H9c2 cells from the Ang II group (P < 0.01, Figure 5A,D ). Compared with the Ang II group, the expression of TAZ observably increased in H9c2 cells from the Ang II + Exo group (P < 0.05).

## Discussion

Cardiomyocyte hypertrophy is one of the most basic pathological characteristics of HF; therefore, intervention of cardiac hypertrophy may become a novel therapeutic method for HF (Shimizu and Minamino, 2016). Previous studies found that MSCs-exosomes exert a significant improvement in HF through anti-inflammation and anti-apoptosis for cardiomyocytes (Chen et al., 2020). We successfully mimicked cell hypertrophy damage to cardiomyocytes by Ang II-induced H9c2 cells. The present study demonstrated that MSCs-exosomes inhibited apoptosis of Ang II-induced H9c2 cells and decrease the levels of BNP (cardiomyocyte damage indicator) and inflammatory factors. These protective effects of MSCs-exosomes on hypertrophic cardiomyocytes may be regulated by Hippo-YAP pathway.

Cardiomyocyte hypertrophy, apoptosis, and inflammation are essential pathological events in HF progression. Several previous studies found that exosomes can regulate apoptosis and inflammation by transporting signal molecules between cells (Guo et al., 2020). Ning et al. (2021) showed that MSCs-exosomes protect myocardial infarction by decreasing pro-inflammatory factors and apoptosis of cardiomyocyte in a myocardial infarction model. Similar with previous studies, our study showed that MSCs-exosomes decreased the expression of pro-apoptotic protein Bax and caspase 3, and the levels of inflammatory factors IL-1β, IL-4, IL-6 and TNF-α in Ang II-induced H9c2 cells. These results confirmed that MSCs-exosomes have anti-apoptosis and anti-inflammation effects on hypertrophic cardiomyocytes in HF.

The Hippo-YAP pathway is one of the main signaling pathways that regulate cell proliferation and apoptosis in various diseases, including HF (Chen and Zhou, 2015; Zhang et al., 2019). YAP and its homolog transcriptional coactivator TAZ are the main downstream genes of the Hippo-YAP pathway (Yan et al., 2019). When the Hippo-YAP pathway is activated, YAP/TAZ are phosphorylated and moved to the cytoplasm and inactivated, while the remaining YAP/TAZ in the nucleus continues to bind with transcription factors to exert regulatory functions (An et al., 2019). Our present results demonstrated that MSCs-exosomes could inactivate Hippo-YAP pathway by suppressing phosphorylation of YAP and increasing the total TAZ expression. It suggests that the Hippo-YAP pathway is involved in the protective mechanism of MSCs-exosomes on HF. Furthermore, it has been confirmed that phosphorylated YAP protein promotes LPS-induced pulmonary micro-vascular endothelial cell apoptosis (Yi et al., 2016). Zheng et al. (2020) indicated that YAP was involved in cardiomyocytes apoptosis by regulating the expression of Bax, Bcl-2 and caspase 3. Taken together, we speculated that the Hippo-YAP pathway might be involved in the anti-apoptosis mechanism of MSCs-exosomes in hypertrophic cardiomyocytes.

In conclusion, MSCs-exosomes inhibited the progression of HF by reducing the apoptosis and inflammation levels of hypertrophic cardiomyocytes. The potential mechanism of MSCs-exosomes alleviating HF may be related with the regulation of the Hippo-YAP pathway. This study confirmed the beneficial effect of MSCs-exosomes for HF treatment. However, what we did is a preliminary exploration about the mechanisms *in vitro*, without the verification of animal experiments. Also, by western blotting, we preliminarily confirmed that MSCs-exosomes alleviate HF by regulating Hippo-YAP pathway, which needed to be explored in more depth.
